# Hyperacute extensive spinal cord infarction and negative spine magnetic resonance imaging: a case report and review of the literature

**DOI:** 10.1097/MD.0000000000022900

**Published:** 2020-10-23

**Authors:** Gianluca Costamagna, Megi Meneri, Elena Abati, Roberta Brusa, Daniele Velardo, Delia Gagliardi, Eleonora Mauri, Claudia Cinnante, Nereo Bresolin, Giacomo Comi, Stefania Corti, Irene Faravelli

**Affiliations:** a Department of Pathophysiology and Transplantation (DEPT), Dino Ferrari Centre, Neuroscience Section, University of Milan; b Foundation IRCCS Ca’ Granda Ospedale Maggiore Policlinico, Neurology Unit; c Foundation IRCCS Ca’ Granda Ospedale Maggiore Policlinico, Neuroradiology Unit, Milan, Italy.

**Keywords:** case report, longitudinally extensive transverse myelopathy, magnetic resonance imaging, spinal cord infarction

## Abstract

**Rationale:**

Spinal cord infarction (SCI) accounts for only 1% to 2% of all ischemic strokes and 5% to 8% of acute myelopathies. Magnetic resonance imaging (MRI) holds a role in ruling out non-ischemic etiologies, but the diagnostic accuracy of this procedure may be low in confirming the diagnosis, even when extensive cord lesions are present. Indeed, T2 changes on MRI can develop over hours to days, thus accounting for the low sensitivity in the hyperacute setting (ie, within 6 hours from symptom onset). For these reasons, SCI remains a clinical diagnosis. Despite extensive diagnostic work-up, up to 20% to 40% of SCI cases are classified as cryptogenic. Here, we describe a case of cryptogenic longitudinally extensive transverse myelopathy due to SCI, with negative MRI and diffusion-weighted imaging at 9 hours after symptom onset.

**Patient concerns:**

A 51-year-old woman presented to our Emergency Department with acute severe abdominal pain, nausea, vomiting, sudden-onset of bilateral leg weakness with diffuse sensory loss, and paresthesias on the trunk and legs.

**Diagnoses:**

On neurological examination, she showed severe paraparesis and a D6 sensory level. A 3T spinal cord MRI with gadolinium performed at 9 hours after symptom onset did not detect spinal cord alterations. Due to the persistence of a clinical picture suggestive of an acute myelopathy, a 3T MRI of the spine was repeated after 72 hours showing a hyperintense “pencil-like” signal mainly involving the grey matter from T1 to T6 on T2 sequence, mildly hypointense on T1 and with restricted diffusion.

**Interventions:**

The patient was given salicylic acid (100 mg/d), prophylactic low-molecular-weight heparin, and began neuromotor rehabilitation.

**Outcomes:**

Two months later, a follow-up neurological examination revealed a severe spastic paraparesis, no evident sensory level, and poor sphincteric control with distended bladder.

**Lessons:**

Regardless of its relatively low frequency in the general population, SCI should be suspected in every patient presenting with acute and progressive myelopathic symptoms, even in the absence of vascular risk factors. Thus, a clinical presentation consistent with a potential vascular syndrome involving the spinal cord overrides an initially negative MRI and should not delay timely and appropriate management.

## 1 Introduction

Spinal cord infarction (SCI) is a rare condition that accounts for only 1% to 2% of all ischemic strokes and 5% to 8% of acute myelopathies. SCI is a clinical diagnosis, but imaging can be useful to rule out other etiologies. Since the pathognomonic T2 changes on magnetic resonance imaging (MRI) can develop over hours to days, the sensitivity of this procedure is low in the hyperacute setting (ie, within 6 hours from symptom onset). Although diffusion-weighted imaging (DWI) holds the potential of identifying spinal cord hyperacute ischemic changes, the sensitivity of this method is imperfect due to the small size of the spinal cord, which increases the likelihood of imaging-related artifacts.^[1,2]^

Longitudinally extensive transverse myelopathy (LETM) refers to a spinal cord lesion extending for 3 or more vertebral segments.^[3]^ Most common causes of LETM include inflammatory myelitis (neuromyelitis optica, neurosarcoidosis, acute disseminated encephalomyelitis, paraneoplastic and postinfectious disorders, HTLV-I, HIV, varicella-zoster virus, and syphilis), neoplasms (ependymoma and other gliomas), nutritional causes (vitamin B_12_ and copper deficiencies) and vascular etiologies (such as arteriovenous malformations, hemorrhages, and ischemic lesions).^[3]^ Despite extensive diagnostic workup, 20% to 40% of SCI cases remain cryptogenic.^[4]^ Here, we present a case of cryptogenic LETM due to SCI with a negative MRI and DWI work-up at 9 hours after symptom onset.

## 2 Case presentation

A 51-year-old woman presented to our Emergency Department with acute severe abdominal pain, nausea, vomiting, sudden-onset of bilateral leg weakness with diffuse sensory loss, and paresthesias on the trunk and legs. After 1 hour, she developed chest tightness with acute urinary retention requiring catheterization. Sensorimotor disturbances reached a plateau after 6 hours. On neurological examination, she showed severe paraparesis and a D6 sensory level. Deep tendon reflexes were absent in the lower limbs and the plantar response was silent bilaterally. Abdominal reflexes were absent. Light touch, pinprick, temperature, and position sense were decreased in the trunk and lower limbs circumferentially.

The patient denied recent traumatic injuries, strenuous physical exertion, or Valsalva maneuver before symptom onset, scuba diving, exposure to radiation or toxic substances, recent infections, and vaccinations. She had a negative history of hypertension, diabetes, smoke, drug abuse, hypercholesterolemia, heart arrhythmias, and recent cardiac or aortic surgery interventions.

In the Emergency Department, complete blood count, coagulation tests, and comprehensive metabolic panel were within normal limits; toxicological screening was negative for recent drug abuse. EKG showed normal sinus rhythm. An aortic computed tomography angiography showed no sign of acute aortic/artery dissection. A 3T spinal cord MRI with gadolinium, ADC maps, and DWI performed at 9 hours after symptom onset did not detect spinal cord alterations. Neuroimaging ruled out the possibilities of disc extrusion, spinal tumors, arachnoiditis, or arteriovenous shunts. Brain MRI was unremarkable. Cerebral spinal fluid (CSF) analysis yielded normal opening pressure, white cell count (3 cells/mL), protein (32 mg/dL), and glucose levels (67 mg/dL). CSF was negative for comprehensive viral Polymerase Chain Reaction panel, bacterial culture, cytology, and oligoclonal bands.

The patient was admitted to our Neurology Unit and she developed paresthesia and weakness in her left hand and forearm and an ipsilateral Horner's syndrome (ptosis, enophthalmos, and miosis).

**Figure 1:**
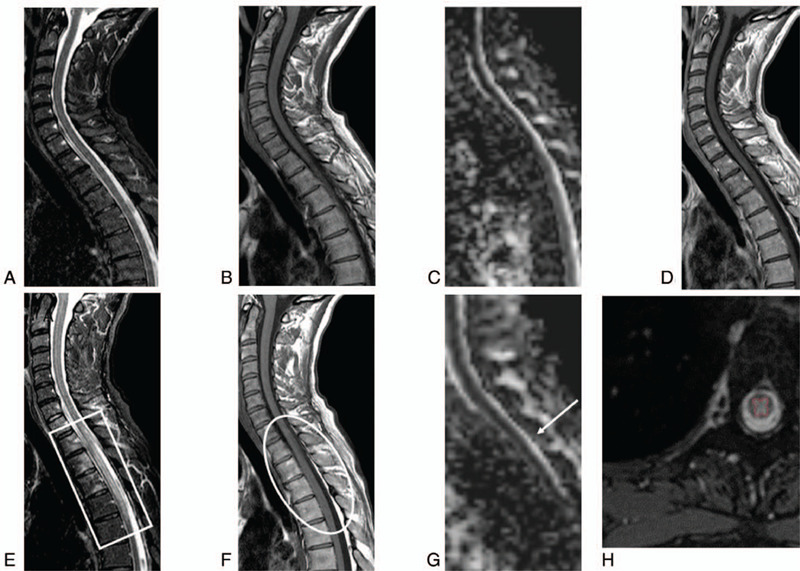
MRI of the spinal cord at 9 and 72 h. MRI images: in the acute phase (A, B, C, D sagittal images; respectively STIR T2, TSE T1, ADC map and TSE T1 post-gad) no spinal cord alterations were seen; the exam was acquired 9 h from the clinical onset. Seventy-two hours later, a new MRI scan (respectively E, F, G sagittal images STIR T2, TSE T1, and ADC map and H axial TSE T2) detected a spinal cord lesion extending from T1-T2 to T6 with a bulgy appearance of the spinal cord (white circle) and a hyperintense signal on STIR T2 images (white rectangle). Moreover, a mild restriction of diffusivity on ADC map, compared to the previous exam (white arrow) and a signal alteration of some vertebral bodies from C7 to T4, more obvious on STIR T2 images as bone marrow edema can be noticed. Axial TSE T2 images (H) showing the prominent gray matter involvement (red line) is suggestive of ischemia rather than myelitis. MRI = magnetic resonance imaging.

Due to the persistence of a clinical picture suggestive of an acute myelopathy, a 3T MRI of the spine was repeated after 72 hours. MRI showed a hyperintense “pencil-like” signal mainly involving the grey matter from T1 to T6 on T2 sequence, mildly hypointense on T1, and with restricted diffusion. The spinal cord showed a mild tumefaction; vertebral marrows from C7 to T4 were hyperintense on T2, presented gadolinium enhancement on post-contrast T1 and restricted diffusion (Fig. 1).

Serologic screening for syphilis, human T-lymphotropic virus 1, varicella-zoster virus, HIV, aquaporin-4, and myelin oligodendrocyte glycoprotein antibodies resulted negative. Other unremarkable blood tests included serum protein electrophoresis, angiotensin-converting enzyme, copper levels, vitamin B12, and erythrocyte sedimentation rate. Analysis of inherited and acquired prothrombotic states comprehensive of antiphospholipid antibodies was negative. Cancer and systemic vasculitis were ruled out by appropriate diagnostic workup. Visual evoked potentials were normal bilaterally.

On the first day of admission, empirical treatment with intravenous acyclovir (10 mg/kg 3 times daily), dexamethasone (8 mg twice daily), and salicylic acid (100 mg/d) was started. We suspended corticosteroid and antiviral therapy once infectious and inflammatory causes were ruled out. In addition, the patient was given prophylactic low-molecular-weight heparin and began neuromotor rehabilitation.

Two months later, a follow-up neurological examination revealed a severe spastic paraparesis, no evident sensory level, and poor sphincteric control with distended bladder.

## 3 Discussion

SCI is an uncommon disease and accounts for only 1% to 2% of all ischemic strokes, but its prevalence increases to 4% to 33% among patients who undergo thoracoabdominal aortic surgery.^[5,6]^ The mechanism of ischemia is multifactorial and involves hypoperfusion due to low-pressure targets to reduce intraoperative hemorrhages, ligation or obstruction of the segmental branch off the aorta and atheroembolism.^[4]^ The peak prevalence of SCI is between the sixth and seventh decade of life^[4]^; clinical presentation includes back or neck pain in up to 70% of patients,^[7]^ which localizes at the level of the infarction, is often radicular and may be transient. However, our patient was younger than average presentation age, had not undergone major surgeries, did not show any vascular risk factors, and presented with no back pain.

The clinical presentation of SCI depends on the transversal and the longitudinal sections of the cord affected. The anterior spinal artery syndrome (ASAS) is the most frequent clinical presentation (Table 1). Less frequent manifestations are classified into a posterior spinal artery syndrome, central cord, and transverse spinal cord syndromes.^[4]^ However, more than one-third of patients have a unilateral pattern or are unclassifiable.^[7]^ In our patient, the acute flaccid paraplegia with a complete sensory loss involving all modalities, the left hand weakness with paresthesia and autonomic dysfunctions with left Horner syndrome, loss of bladder and bowel control can be ascribed to a cervicothoracic partial transverse SCI. Longitudinally, the lesion expanded from lower cervical (Horner syndrome) to middle thoracic segments (paraplegia); transversally, the alteration involved predominantly the left hemicord (weakness of left hand). Although impairment of all sensory modalities is not common in ASAS, an anterior spinal artery (ASA) pattern seems most probable in our patient. The spinal cord presents a complex and highly variable vascularization among individuals. Three branches of the vertebral arteries, the ASA and 2 posterior spinal arteries, vascularize the most proximal part of the spine along a rostro-caudal axis. An intricate supply of cervical and aortic segmental branches, which enter the intervertebral foramina and divide into the anterior and posterior radiculomedullary arteries, contribute to the vascularization of ASA and posterior spinal arteries. In our case, the high interindividual variability of spinal cord vascularization^[8]^ may involve an anatomical variant of ASA Supplying part of the dorsal columns and giving reason to the clinical presentation and the extensive transversal spine involvement on MRI.

**Table 1:**
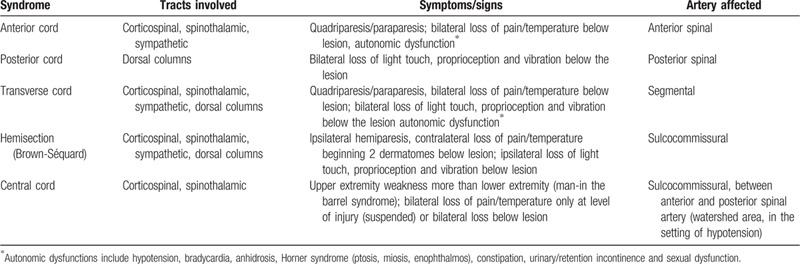
Vascular spinal cord syndromes.

MRI is the preferred imaging modality for the spinal cord. It can display SCI-related alterations such as the owl's sign (inconstant bilateral hyperintensities of the anterior horns on axial T2-weighted images),^[9]^ and longitudinal “pencil-like” T2-weighted hyperintensities with associated cytotoxic edema on sagittal sequences. However, compared with brain imaging, spine MRI is burdened by important limitations. Indeed, the bone enclosing the spine causes magnetic field distortions and generate artifacts.^[10]^ Although researchers have developed techniques to compensate for artifacts,^[11]^ the effects of motion distortion caused by respiration, CSF, and arterial pulsation, as well as swallowing can result in distorted images.^[10]^ Moreover, compared to 1,5T scanners, higher field strength in 3T MRI scanners can produce artifacts in spinal cord imaging due to magnetic field inhomogeneity.^[12]^ Another inconvenience of current MRI imaging of the spinal cord is the limited spatial resolution.^[4]^ Indeed, the clinical presentation of ASAS may resemble a central cord lesion on MRI,^[4]^ such as in our patient. Moreover, even extensive lesions may be missed on the initial MRI and then detected on repeated imaging (Table 2).

**Table 2:**
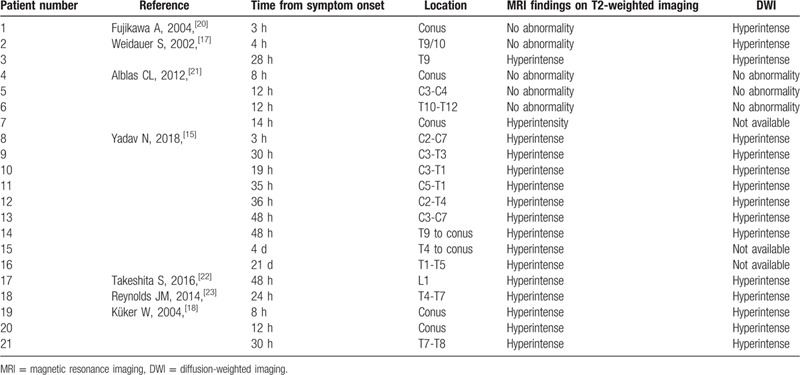
Summary of magnetic resonance imaging findings on T2-weighted imaging and diffusion-weighted imaging in previously reported patients with spinal cord ischemia.

Spinal cord alterations on T2-weighted imaging such as “pencil-like” lesions and the owl's sign alone usually do not provide enough specificity to make a diagnosis of SCI. However, radiological signs can sometimes provide diagnostic clues. For example, vertebral body infarction indicated as abnormal bone marrow signal on T2 sequences can be associated with SCI. The prevalence of vertebral body infarction sign varies among studies.^[12–14]^ This sign can appear after eight hours from onset of the symptoms up to a few days later and its prevalence ranges from 14% to 44% in researches based on various SCI-related etiologies.^[13]^ Although some analyses indicate a significant association of vertebral body hyperintensity with SCI related to aortic pathology^[13]^ and arterial embolism,^[12]^ this sign can be observed in infections, fractures, and metastasis.^[14]^ Vertebral body infarction may not always present with SCI because arterial occlusion may localize distal to the vessels supplying the vertebral body or because of the adequate collateral blood supply of the vertebral bone.

In addition to radiological signs associated with SCI on T2-weighted imaging, DWI has improved the characterization of a wide range of diseases of the spinal cord, including SCI.^[11]^ The shortest time reported between the onset of the symptoms of SCI and DWI abnormalities is 3 hours.^[15,16]^ Some studies evaluating MRI imaging of SCI show DWI abnormalities as early as 4 hours^[17]^; in other patients, DWI alterations at 8 and 12 hours were associated with T2 hyperintensities.^[18]^ Hence, spinal cord MRI with the addition of DWI can help in diagnosing SCI early in the disease course. However, no large studies have established a precise time threshold for diffusion changes in SCI patients.^[15]^ Moreover, despite the use of techniques to reduce spatial distortion and improve the quality of the images,^[11]^ some technical challenges persist for DWI of the spinal cord. These include the small size of the spine compared to the brain as well as respiratory and cardiac motion artifacts.^[11]^ Although SCI is a clinical diagnosis, these limitations can decrease the sensitivity of spinal cord MRI even in cases with extensive spinal cord lesions and may lead to an important delay in patient's diagnostic workup and treatment.

Despite the potentially devastating clinical outcomes, no large-scale studies have evaluated the effect of different therapies on SCI.^[19]^ This is mainly due to the low incidence of the disorder and the frequent delay in diagnosis. Therefore, the quality of evidence for every treatment in SCI is currently weak. Based on recommendations for acute treatment of stroke at any site, the current consensus recommends salicylic acid as a prudent first choice drug.^[19]^ Experience with thrombolysis comes from case reports^[4]^ and its use is contraindicated in patients with recent major surgery and aortic dissection. Corticosteroids are indicated for patients with vasculitis. The use of vasopressors in case of hypotension, as well as lumbar CSF drainage to reduce counterpressure and increase spinal cord perfusion, have been associated with remarkable improvements in some patients.^[4]^ Unfortunately, these approaches are not supported by large trials. Currently, no compelling evidence suggests that therapeutic hypothermia improves clinical outcomes in patients with SCI.^[4]^ However, all patients with SCI should be monitored for hypoglycemia and hyperglycemia, deep vein thrombosis, pulmonary embolism, urinary tract infection, and pressure ulcers.

## 4 Conclusions

Regardless of its relative infrequence, SCI should be suspected in every patient with acute and progressive myelopathic symptoms, even without associated vascular risk factors. MRI is important in ruling out other causes and in diagnosing SCI. However, sensitivity and specificity of spine MRI with T2-weighted imaging and DWI may be imperfect in confirming the diagnosis, even with extensive spinal cord lesions and particularly in the hyperacute setting. Thus, the patient's history and careful examination suggesting a potential vascular syndrome involving the spinal cord override an initially negative MRI and should not delay timely and appropriate management.

## Acknowledgment

The authors gratefully thank the Associazione Centro Dino Ferrari for its support.

## Author contributions

Gianluca Costamagna: Study concept and design, major role in the acquisition of data, analysis and interpretation of data, drafting and revising the manuscript; Megi Meneri: study concept and design, major role in the acquisition of data, drafting and critical review of the manuscript; Elena Abati: drafting and critical review of the manuscript, major role in the acquisition of data; Roberta Brusa, Daniele Velardo, Delia Gagliardi, Eleonora Mauri: major role in the acquisition of data; Claudia Cinnante: analysis and interpretation of imaging data; Nereo Bresolin, Giacomo Comi: study supervision; Stefania Corti and Irene Faravelli: interpretation of data, drafting and revising the manuscript, study supervision.
